# *TOMM40* genetic variants associated with healthy aging and longevity: a systematic review

**DOI:** 10.1186/s12877-022-03337-4

**Published:** 2022-08-13

**Authors:** Sunny Chen, Sara M. Sarasua, Nicole J. Davis, Jane M. DeLuca, Luigi Boccuto, Stephen M. Thielke, Chang-En Yu

**Affiliations:** 1 Geriatric Research, Education, and Clinical Center, Puget Sound VA Medical Center, VA Puget Sound Healthcare System, 1660 S Columbian Way, Seattle, WA 98108 USA; 2 Healthcare Genetics Program, School of Nursing, Clemson University, Clemson, SC USA; 3 Department of Psychiatry and Behavioral Sciences, University of Washington School of Medicine, Seattle, WA USA; 4 Department of Medicine, Division of Gerontology and Geriatric Medicine, University of Washington, Seattle, WA USA

**Keywords:** Aging, Healthy aging, Systematic review, TOMM40, Longevity

## Abstract

**Introduction:**

Healthy aging relies on mitochondrial functioning because this organelle provides energy and diminishes oxidative stress. Single nucleotide polymorphisms (SNPs) in *TOMM40*, a critical gene that produces the outer membrane protein TOM40 of mitochondria, have been associated with mitochondrial dysfunction and neurodegenerative processes. Yet it is not clear whether or how the mitochondria may impact human longevity. We conducted this review to ascertain which SNPs have been associated with markers of healthy aging.

**Methods:**

Using the PRISMA methodology, we conducted a systematic review on PubMed and Embase databases to identify associations between *TOMM40* SNPs and measures of longevity and healthy aging.

**Results:**

Twenty-four articles were selected. The *TOMM40* SNPs rs2075650 and rs10524523 were the two most commonly identified and studied SNPs associated with longevity. The outcomes associated with the *TOMM40* SNPs were changes in BMI, brain integrity, cognitive functions, altered inflammatory network, vulnerability to vascular risk factors, and longevity.

**Discussions:**

Our systematic review identified multiple *TOMM40* SNPs potentially associated with healthy aging. Additional research can help to understand mechanisms in aging, including resilience, prevention of disease, and adaptation to the environment.

## Introduction

In a recent report entitled “GHE: Life expectancy and healthy life expectancy,” the World Health Organization (WHO) confirmed that global life expectancy has increased by more than 6 years from 66.8 to 73.4 years in the past two decades [1]. However, the report also suggested that the Healthy Life Expectancy (HALE) did not increase proportionally with life expectancy; and on average, people lived with some form of disability for 9.7 years before death [1]. Bodily deterioration and increased susceptibility to diseases and medical conditions are inevitable parts of aging. The topic of longevity is no longer as simple as living long, but should also focus on the average length of a healthy life [2].

In the recent past, longevity studies have shifted to the study of phenotypic age. These studies proposed methods to identify reliable genetic biomarkers such as DNA methylation and leukocyte telomere length [3, 4] or exonic and non-coding single nucleotide polymorphism (SNP) variants [5] as surrogate measures to assess an individual’s healthy life expectancy. Many of these genetic factors were tested on mouse models; and the results suggested a close interconnection between genome maintenance and the underlying biological mechanisms of aging [6]. In 2014, a genome-wide association meta-analysis identified a novel locus containing the genes *TOMM40*/*APOE*/*APOC1* that were linked to human longevity [7].

The *TOMM40* gene maps on chromosome 19q13.32 and encodes the TOM40 protein, which is a subunit of the Translocase of Mitochondrial Outer Membrane (TOM) complex [8]. TOM is essential for mitochondrial functions including energy metabolism, cell apoptosis, lipid synthesis, and cellular homeostasis [9]. Several genetic association studies have revealed a strong link between genetic variants in multiple loci and exceptional human longevity [10, 11]; and *TOMM40* was identified as one of the candidate genes. However, it is still unclear how *TOMM40* variants directly influence the pathway of aging.

As the powerhouse of the cell, the function of mitochondria determines the efficacy of metabolic and signaling pathways. Recent studies demonstrated that elevated *TOMM40* mRNA expression is associated with fold changes in mitochondria membrane potential (Δψm), mitochondrial DNA (mtDNA) copy number, respiratory rate, and ATP production [12, 13]. Mitochondrial functions are tightly regulated, and their dysfunctions can be associated with disease pathogeneses. For instance, elevated Δψm is observed in most tumors [14], while a decrease Δψm is linked to decreased ATP production rate, ATP depletion, and metabolic acidosis [15]. A decrease in mtDNA copy number is significantly associated with frailty (OR 0.91, 95% CI, 0.85–0.97), and is a strong predictor of all-cause mortality in age- and sex-adjusted multi-ethnic populations with a pooled hazard ratio of 1.47 (95% CI, 1.33–1.62) [16]. Impaired mitochondrial respiratory rate and decreased oxygen metabolism were associated with traumatic brain injury [17].

A recent review article suggested that mitochondrial dysfunction plays critical role as both primary and secondary events in Alzheimer’s disease (AD) pathogenesis through impaired energy metabolism, increased oxidative stress, and disturbed genomic homeostasis [18]. Research has investigated the association between *TOMM40* SNPs and the development of AD. For instance, the poly-T length of SNP rs10524523 can increase the risk of AD [19–21] and the rs2075650 SNP was shown to be associated with higher AD risk in Asian and Caucasian populations [22]. Yet recent studies have revealed that mitochondria dysfunction can also lead to non-AD cognitive decline associated with aging in healthy individuals [12, 13, 23]. These findings suggested that there are non-pathogenic *TOMM40* variants that may contribute to the pathways linked to longevity.

This review aims to investigate the possible connections between *TOMM40* SNPs and longevity, independent of disease or medical conditions. In order to develop a deeper understanding of *TOMM40* genetic variants in individuals without dementia, we conducted a systematic review of published research studies that examined the role of *TOMM40* SNPs in the aging process. Since the TOM40 protein is a key component of mitochondria [24], it has been hypothesized that variants in the corresponding gene can be associated with improved or reduced mitochondrial functions. Such differences may lead to biological changes in aging. The goals of this systematic review were to: 1) identify the top candidate SNPs that are commonly identified in the studies, and 2) elucidate the associated age-related phenotype, and the specific allele that is associated with longevity and the aforementioned phenotypes. We sought to fill in the gap of knowledge by defining what is currently known about *TOMM40* in healthy aging, and help shape directions for future research studies.

## Methods

The peer-reviewed articles included in this systematic review were identified from the PubMed and Embase databases. We identified all studies that are associated with *TOMM40*, aging, and longevity. We conducted the review based on the guidelines of the PRISMA statement [25].

### Conceptualization of aging

Aging entails a variety of phenotypic changes in cognitive function, frailty, metabolism, muscle-skeleton, height & weight, and vulnerability. Healthy aging involves the ability of the body to maintain proper mental and physical capability through these changes, and enables wellbeing in older age [26]. These capabilities are referred to the ability to meet their basic needs, to make decisions, to be mobile, to maintain relationships, and to contribute to the society [27]. Because there is no single physiologic parameter that covers all these domains, we used a broad approach in identifying research.

### Article selection

The initial PubMed search terms used two combinations of (TOMM40[All Fields] AND AGING[All Fields]) and (TOMM40[All Fields] AND LONGEVITY[All Fields]). The search was conducted on all publications published before the 11th of March, 2022. We did not limit the search by the publication language. The same search terms were conducted in Embase for verification, and we combined the results from the two searches.

We manually reviewed the articles to identify the effect of *TOMM40* genetic variants on healthy aging. Due to *TOMM40* being commonly connected with AD; we excluded the studies that were solely focused on the prediction of dementia outcomes, and studies specifically included patients with AD diagnoses. Current RNA quantification methodologies have limited capability to distinguish between *TOMM40* mRNA and its pseudogenes [13]. Due to this reason, studies that focused purely on *TOMM40* RNA may generate contradicting results. Thus, studies on *TOMM40* RNA and TOM40 protein levels that did not include SNP data were excluded from the review. We excluded articles that used non-human models for investigation.

### Data extraction

We summarized the articles by extracting the information including author, year of publication, SNP accession numbers (rs ID) investigated in the study, and proposed phenotypes associated with the SNPs. We also listed all the SNPs of the neighboring genes (including *PVRL2, APOE,* and *APOC1*) that are co-investigated from each study. We have noted the specific variants that were tied to the associated phenotype unless it is not specified in the study. For the interest of investigation into the SNP effect on aging, we carefully extracted only age-associated phenotypes (including nonpathogenic changes associated with BMI, cognitive function, and vulnerability) from each study. Additionally, we compiled a list of the most commonly identified *TOMM40* SNPs and ranked them based on the number of appearances in different studies.

## Results

**Fig. 1:**
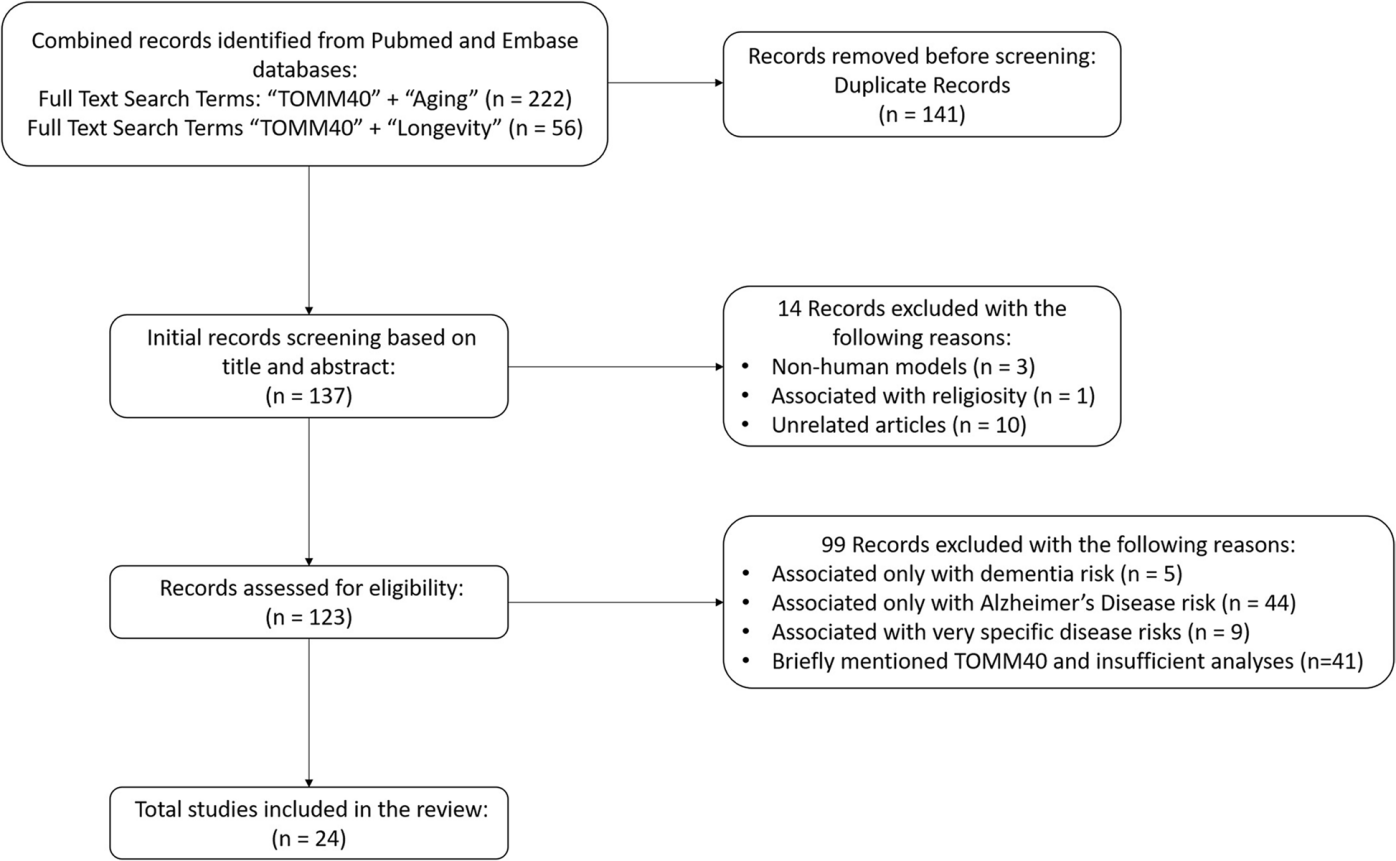
PRISMA flowchart of article selection

The initial search identified 123 articles. By screening through the titles and abstracts of the articles and evaluating the results, we identified 24 studies that met the inclusion and exclusion criteria to be included in the review. Figure 1 presents the flowchart that describes the literature selection process.

All of the research studies were published between the years 2011 and 2022. The number of *TOMM40* SNPs investigated in each study varied widely, ranging from one to ten. Half of the studies were exploratory studies such as Genome-Wide Association Studies (GWAS) and SNP panels [7, 10–, 28–35]. The remaining studies [36–, 37–49] specifically selected a *TOMM40* SNP or SNPs of interest. Sixteen unique SNPs were investigated in the studies. In addition to the *TOMM40* SNPs, eight studies investigated polymorphisms of neighboring genes of *TOMM40* including *APOE, APOC1,* and *PVRL2*. The *APOE* SNPs rs429358, rs405509, and rs769449 are the most common polymorphisms that are investigated and reported together in the studies. Two of these papers included *PVRL2* SNPs in the analyses [29, 45], and two papers investigated *APOC1* SNPs [29, 40]. The 4 most common phenotypes studied were: changes in Brain Integrity, Cognitive Ability, Longevity, and BMI. Table 1 summarizes the results of the systematic review.

**Table 1:** Summary of the articles investigating *TOMM40* SNPs and aging or longevity

Authors	Year	Cohort Ancestry	Study Type	SNPs rs Number and Allele Associated with the Phenotype				***TOMM40***-Associated Phenotype Summary
				***PVRL2***	***TOMM40***	***APOE***	***APOC1***	
Li [49]	2022	European	Selected Single SNP		rs2075650-g			Led to less language comprehension network strength in females
Gui [36]	2021	Chinese	Selected Single SNP	N/A	rs2075650-g	N/A	N/A	Increased vulnerability to global cognitive decline due to: smoking, drinking, physical inactivity, obesity, total cholesterol (include LDL), triglycerides, diabetes, and hypertension
Liu [10]	2021	Chinese	SNP Array	N/A	rs2075650-A	N/A	N/A	Increased longevity
Deters [37]	2021	Non-Hispanic Black & Non-Hispanic White	Selected Single SNP	N/A	rs10524523-S	N/A	N/A	Altered age-related global cognition, episodic memory, and visuospatial ability segregated by *APOE* variant: *TOMM40* SNP in *APOE* ε3/ε3: Faster decline *TOMM40* SNP in *APOE* ε4+: Slower decline
Lamparello [28]	2020	Conducted in the United States^b^	SNP Array	N/A	rs2075650-g	N/A	N/A	Altered inflammation response (increased severity) in age-associated blunt injuries
Kulminski [38]	2019	Caucasian	Selected Multiple SNPs	N/A	rs2075650-A/g	rs429358-C	N/A	Lower BMI
Yashin [29]	2018	Mixed Population (World-Wide)	GWAS	rs73052307-T	rs2075650-A rs71352238-T rs34095326-G rs157582-G	rs769449-G	rs56131196-G	Increased longevity
Yashin [30]	2018	Mixed Population (World-Wide)	GWAS	N/A	rs2075650-A rs8106922-A rs157582-C rs71352238-T	rs405509-G rs769450-G rs769449-G	N/A	Increased longevity
Arpawong [31]	2017	Conducted in the United States^b^	GWAS	N/A	rs71352238-G rs2075650-g rs157582-A	rs769449-a	N/A	Delayed verbal recall in non-AD
Burggren [39]	2017	Conducted in the United States^b^	Selected Single SNP	N/A	rs10524523-L	N/A	N/A	Thinner entorhinal cortex in non-AD
Shadyab [40]	2017	Conducted in the United States^b^	Selected Multiple SNPs	N/A	rs2075650	rs429358	rs4420638	Increased longevity in women
Lin [41]	2016	Chinese	Haplotype	N/A	rs7254892-A rs157580-A rs2075649-A rs2075650-A rs157582-T rs8106922-A rs1160985-T	rs405697-G^a^ rs439401-C^a^ rs445925-A^a^	N/A	Increased longevity
Payton [42]	2016	European	Selected Single SNP	N/A	rs10524523-S	N/A	N/A	Slower vocabulary ability decline in non-AD
Wennberg [43]	2016	Conducted in the United States^b^	Selected Single SNP	N/A	rs10524523	N/A	N/A	No association with cortical thinning in non-AD
Deelen [7]	2014	European	GWAS	N/A	rs2075650	rs4420638-A	N/A	Increased longevity
Greenbaum [44]	2014	Israeli Jewish	Selected Single SNP	N/A	rs10524523-S	N/A	N/A	Better cognition in non-AD
Lu [45]	2014	Chinese	Selected Multiple SNPs	rs12972156-A rs519825-A rs395908-C	rs2075650-A/g	rs405509-A	N/A	Increased longevity
Ferencz [32]	2013	European from Island of Kungsholmen	SNP Array	N/A	rs11556505 rs2075650	N/A	N/A	No association with hippocampal volume and episodic memory performance in non-AD
Zhang [35]	2013	Conducted in the United States^b^	GWAS	N/A	rs115881343	rs769449	N/A	Cognitive decline in non-AD
Caselli [46]	2012	Conducted in the United States^b^	Selected Single SNP	N/A	rs10524523	N/A	N/A	Age-related memory performance in non-AD
Guo [33]	2012	Mixed Population (World-Wide)	GWAS	N/A	rs2075650-g	N/A	N/A	Low BMI
Sebastiani [34]	2012	Conducted in the United States^b^	GWAS	N/A	rs2075650-A	N/A	N/A	Increased longevity
Maruszak [47]	2012	European	Selected Single SNP	N/A	rs10524523-L	N/A	N/A	Decreased longevity
Johnson [48]	2011	Conducted in the United States^b^	Selected Single SNP	N/A	rs10524523-VL	N/A	N/A	Cognitive decline in non-AD

rs#: Accession number for specific SNPs

*BMI* Body Mass Index

*non-AD* Non-Alzheimer’s disease patients

*N/A* Not available

^a^ Located at *APOE*-to-*APOC1* intragenic region

^b^ Studies conducted in the United States with mixed population

### Brain integrity and cognitive ability

Nine studies investigated the effect of *TOMM40* SNPs on cognitive ability among individuals without AD [31, 32, 35, 37, 42, 44, 46, 48, 49]. The evaluations differed across research studies but were mainly focused on episodic memory performances and verbal/vocabulary abilities. Although there are inconsistent findings, five studies reported that the SNP rs10524523 is associated with cognitive decline [37, 42, 44, 46, 48]. Deters et al. suggested that the effect could be attenuated or exacerbated due to concomitant *APOE* variants in non-Hispanic Black populations [37]. Thus, the impact of *TOMM40* may be affected by the presence of other risk factors and may contribute to inconsistent reports. One study done by Ferencz et al. reported no association between *TOMM40* SNP and cognition [32].

Two studies examined the effect of *TOMM40* SNPs on brain integrity (in the form of thinning of different brain regions) using brain imaging. These studies hypothesized that there would be phenotypic changes in the brain of non-AD individuals. Of the two studies, Burggren et al. found an association between SNP rs10524523 and entorhinal cortex thinning [39], while the other study reported no association between *TOMM40* SNPs and cortical thinning/hippocampal volume [43].

### Longevity

All nine studies that investigated associations between *TOMM40* SNPs and longevity found at least one SNP associated with human longevity [7, 10, 29, 30, 34, 40, 41, 45, 47]. Three studies presented their findings as *TOMM40* haplotype data instead of a single SNP analysis. Among the SNPs inspected, the association of the rs2075650-A variant with increased longevity is consistent across multiple studies. This finding was consistent for the American, European, and Chinese cohorts.

### Body mass index

Two studies found an association between one single *TOMM40* SNP (rs2075650) with Body Mass Index (BMI) status. Guo et al. reported that being homozygous at rs2075650-g allele is associated with lower BMI [33]. While Kulminski et al. suggested that only heterozygous in A/g is associated with lower BMI [38].

### Other factors

Two studies reported that the rs2075650-g allele can alter human inflammatory response and susceptibility to diseases. Lamparello et al. found a significant association between higher severity in traumatic injury due to predisposition to anti-inflammatory response with rs2075650-g allele [28]. The author suggested a differential inflammation profile in patients who carried the g allele. Such inflammatory predisposition can lead to greater disease severity and a higher mortality rate in the elderly. Similarly, Gui et al. suggested that the genetic effect of rs2075650-g allele aggregates with vascular risk factors (VRF), and increases the vulnerability to global cognitive decline [36].

Table 2 summarizes the most commonly investigated *TOMM40* SNPs and the associated health factors across all the studies. The most commonly reported and investigated polymorphism is rs2075650 – included in 11 different studies. SNP rs10524523 is ranked number 2, and has appeared in eight studies. The SNPs rs157582, rs71352238, and rs8106922 were specific to longevity, and were usually investigated together: rs157582 has been examined in 4 studies, 3 times for rs71352238, and 2 for rs8106922.

**Table 2:** Top 5 Commonly Investigated *TOMM40* SNPs From Studies and Their Association with Age-Related Health Factors and Longevity

Common ***TOMM40*** SNPs	Times Identified	Associated Age-related Health Factor and/or Longevity
rs2075650	12	BMI [33, 38]; Cognitive Function [31, 49]; Longevity [7, 10, 29, 30, 34, 40, 41, 45]; Cognitive Function^a^ [32]; Altered Inflammatory Network [28]; Vulnerability to Vascular Risk Factors [36]
rs10524523	7	Brain Integrity [39]; Cognitive Function [37, 42, 44, 46, 48]; Longevity [47] Brain Integrity^a^ [43]
rs157582	4	Longevity [29–31, 41]
rs71352238	3	Longevity [29–31]
rs8106922	2	Longevity [30, 41]

*BMI* Body Mass Index

^a^Investigated, but no direct association found between the SNPs and the health factor

## Discussion

This systematic review identified the most commonly studied *TOMM40* SNPs and the age-associated health factors that were investigated for these genetic variants. The studies we identified indicate that three main age-related health factors may be associated with *TOMM40* SNPs: brain integrity, body mass index, and cognitive function. Brain integrity is associated with SNP rs10524523. BMI is associated with SNP rs2075650. Both SNPs are also related to cognitive function in non-AD individuals. Additionally, all of the *TOMM40* SNPs listed in Table 2 are associated with human longevity. We will discuss the potential mechanisms for each of these.

It is uncertain why the non-AD aging-associated phenotypes of the gene *TOMM40* have only been investigated since 2011, the date of the earliest publication. However, Roses et al.’s 2016 study have pointed out that early GWAS studies have deliberately excluded the analyses of the *APOE*-*TOMM40* linkage disequilibrium (LD) region, which resulted limited understanding in the *TOMM40* gene [50]. Additionally, due to strong LD between the *APOE* and *TOMM40* SNPs, many strong GWAS signals for *TOMM40* SNPs were interpreted as *APOE* hits in early research studies [32]. Early interpretations of the gene *TOMM40* may discourage research groups from investigating into it; and may explain why topics in *TOMM40* and non-AD aging have only be investigated after 2011. Aside from longevity, the most commonly investigated aging-associated phenotype is brain function. It is to be expected that brain integrity and cognition receive so much attention, as the mitochondrial cascade and mitochondrial dysfunction have become some of the most predominant hypotheses in AD studies [51, 52].

### SNP rs2075650

**Fig. 2:**
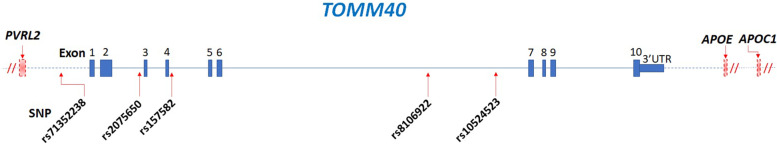
Top *TOMM40* SNPs Found to be Associated with Longevity. The data of *TOMM40* gene and *TOMM40* SNPs locations referenced from UCSC Genome Browser [56], accessed on 11th March, 2022. The boxes indicate the ten exons of *TOMM40*. The dotted boxes indicate the neighboring genes: *PVRL2*, *APOE*, and *APOC1*. The // marks indicate that the neighboring genes are not fully displayed in the figure. The solid lines indicate the introns of *TOMM40*. The dotted lines indicate the 3′ and 5′ intragenic region of *TOMM40*

The rs2075650 SNP (depicted in Fig. 2) is located within the non-coding region of *TOMM40*: c.275-31A > G [53]. The minor allele frequency (MAF) for g is approximately 0.124; the frequency is more common in the European (Mean MAF: 0.131) and African populations (Mean MAF: 0.141), and less common in East Asian populations (Mean MAF: 0.092) [54, 55]. This SNP is strongly associated to exceptional longevity [7, 10, 29, 30, 34, 40, 41, 45]. Multiple studies have reported that the major allele rs2075650-A has highly significant (*p* < 0.001) predictability of survival to ≥90 years old in Chinese [41, 45], European [7], and admixed (White, Black, and Hispanic) populations [29, 34, 40]. Its minor allele rs2075650-g is associated with various biological changes including weakened cognitive function [31, 49], decreased BMI [33, 38], altered inflammatory networks [28], and increased vulnerability to vascular risk factors [36].

Two reviewed articles pointed out that the rs2075650-g allele is linked to weakened verbal ability in non-AD individuals. Arpawong et al. reported an association with delayed verbal recall ability [31]; and Li et al. reported an overall decrease in language comprehension network strength in the female population, correlated with increasing age [49]. It has also been suggested that rs2075650 has a strong association with AD, with an odds ratio of 4.178 (*p*-value = < 0.001) in Asian and Caucasian populations [22]. Another meta-analysis found similar associations with AD in European and Korean populations but was unable to replicate the result in the Chinese population [57]. There was one study that demonstrated that the rs2075650-g allele is linked to executive dysfunction and lifetime depression [58]. The underlying biological mechanism behind this SNP variant is still unclear, however, it is known that it has a negative effect on both AD and non-AD populations. A recent study that proposed an accurate surrogate quantification of *TOMM40* transcripts did not find any evidence that the minor allele can affect mRNA levels in both AD and non-AD brain tissues [13]. This suggested that the function of the SNP is not as straightforward as it might seem to be, and may belong to a more complicated genetic regulatory network.

Homozygosity of the g allele is also associated with a lower BMI score [33]. A 1994 review article that proposed an association between AD and weight loss [59], thus we hypothesized that the relationship between low BMI and impaired cognition may be linked to the SNP. Meta-analyses have shown that unintentional weight loss occurs commonly in the elderly [60], and have pointed out that it has a significant association with all-cause mortality in the elderly [61, 62]. These findings suggest that BMI maintenance is an important factor in healthy aging, and may be indirectly mediated by this *TOMM40* SNP.

Two studies have pointed out that the minor allele rs2075650-g altered biochemical pathways can lead to increased susceptibility to bodily decline and disease severity in the elderly [28, 36]. In 2021, Gui et al. reported that vascular risk factors (such as current smoking, current drinking, physical inactivity, obesity, total cholesterol, triglycerides, low-density lipoprotein cholesterol, high lipoprotein cholesterol, diabetes, and hypertension) and rs2075650-g variant could interact synergistically [36]. The g allele increased an individual’s susceptibility to bodily damages due to the aforementioned vascular risk factors, and lead to decline in cognitive performance. Mitochondrial functional variation due to the rs2075650-g allele may provide an explanation for the connections in weight loss, cognitive decline, and vulnerability to higher disease severity. Multiple studies demonstrated that individuals who are homozygous in the A allele, and heterozygous in A/g alleles have longer lifespans [29, 30, 34, 45]. This makes sense as being free of diseases often equates to a prolonged lifespan. However, we are unable to directly ascertain the causal relationship between the *TOMM40* genetic variants and increased lifespan through the studies. It is still unclear if these individuals lived longer because the alleles made them more resilient to weakening due to the aging process; or because the alleles are associated with lesser disease risks.

### SNP rs10524523

Different from other *TOMM40* SNPs, rs10524523 (depicted in Fig. 2) is a poly-T extension that consists of Short (S), Long (L), and Very Long (VL) variants located within the intron 6: c.644-938_644-904 T(12_46) [21]. The findings from the reviewed article suggested that in AD patients, the L variant is associated with higher AD risk and increased cognitive decline in *APOE* e4 carriers [21, 47]. While the VL variant is associated with earlier disease onset in *APOE* e3/e3 carriers [63]. The non-AD studies reviewed in this article suggested a consistent finding of L and VL variants associated with a higher rate of cognitive decline independent of *APOE* isoform. These findings hold true in non-Hispanic Black *APOE* e4 carriers as well [37].

As an intronic gene variation, it is highly unlikely that the rs10524523 SNP may disrupt protein folding that can significantly affect the stability of the TOM complex. However, the reviewed articles pointed out a close association of the poly-T SNP with different forms of brain and cognitive decline [37, 42, 44, 46, 48]. This suggested that longer variants of rs10524523 are linked to certain biochemical cascades that could increase the brain’s vulnerability to environmental insults.

In a recent study, Lee et al. correlated *TOMM40* RNA level with the mitochondrial functions (including mtDNA copy number and membrane potential) of human cell lines in response to oxidative stress [13]. The study reported that after H_2_O_2_ treatment, *TOMM40* RNA levels were increased by 1.2 to 1.5-fold, mitochondrial DNA copy number was decreased by 40–80%, and membrane potential was decreased by 25–80% compared to untreated cell cultures [13]. Their findings suggested that upregulation of *TOMM40* RNA level is associated with mitochondrial dysfunction or its response due to stress; and that it may be a biochemical mechanism for cells to cope with damages to the cells. Therefore, different SNP variations in *TOMM40* may contribute to the overall efficacy gene expressions and transcriptional regulations in response to damages. The biological effect of the length in rs10524523 is currently unknown; however, studies have suggested that the VL variant is associated with a significantly higher *TOMM40* mRNA expression level, as compared to the S variant in both control and AD groups [13, 64]. As noted above, the rs10524523–VL variant is associated with cognitive decline. A possible explanation of such observation is that cognitive decline due to aging is associated with mitochondrial dysfunction. Alzheimer’s disease may be an extreme form of the aging process that resulted from a weaker mitochondria complex coupled with other genetic factors.

### Additional *TOMM40* SNPs

Multiple studies have pointed out additional SNPs including rs71352238 (c.-245 T > C), rs157582 (c.435 + 33C > T), and rs8106922 (c.644-2321A > G) that are linked to increased lifespan. A single GWAS study also showed SNPs rs71352238 and rs157582 are also associated with delayed verbal recall [31]. However, due to limitations in research methods and limited evidence of their association with other medical conditions, their causal relationship with longevity and healthy aging can only be speculated. These polymorphisms, in sequential order, are located at the 5′ upstream region, intron 4, and intron 6 of *TOMM40* (depicted in Fig. 2). Including rs2075650 and rs10524523, the top five SNP candidates associated with aging and longevity are all located in non-coding regions. Two possible reasons may explain the potential biological importance of variances in intronic SNPs.

First, the TOM complex is essential for the recognition and importation of proteins into the mitochondria [8, 24]. Therefore, the mitochondrial complex does not tolerate other TOM40 protein isoforms or misfolding resulting from amino acid changes. There are currently no studies that have reported any SNPs within the exon regions of *TOMM40* that would translate into different functional protein variants in humans. In 2007, Kinoshita et al. reported a protein variant TOM40B with 28 amino acid deletion at C-terminus, that would form a larger protein complex in a rat model [65]. This opens up the possibility of seeing functionally truncated TOM40 isoforms in humans. A UniProt search of the gene revealed that there are two human isoforms; one canonical, and another isoform due to alternative splicing [66]. In 2011, Mager et al. successfully characterized two human TOMM40 protein isoforms that have deletions in their N-terminus [67]. However, the authors did not observe any functional differences that could imply any biological significance [67]. It is also unknown if there are SNPs that contribute to the expression of the truncated isoforms.

Second, multiple regulatory elements can interact with the non-coding regions of the *TOMM40* gene, and the SNPs can alter mRNA expression by affecting the binding affinities. Two earlier studies have suggested that the rs10524523-VL variant is associated with a higher expression level of *TOMM40* mRNA and TOM40 protein, and the increased expression is protective against the toxic effects of exogenous beta-amyloid (Aβ) [12, 64]. However, a meta-analysis conducted in 2015 concluded that rs2075650 can also affect *TOMM40* expression level, and over-expression can lead to the accumulation of amyloid precursor protein [57]. It is not known why both SNPs that can lead to over-expression of RNA and protein level can have a very different biological effect. This suggested that these SNPs may also play different roles in other regulatory pathways that have yet to be properly examined. For instance, one study has shown that *TOMM40* can be regulated by long non-coding RNA (lncRNA) [68]. It is unknown if the gene itself produces any non-coding RNAs, but it is possible that intronic SNPs may alter the transcription of both functional and non-function RNA products. Another example is pseudogene regulation. A study reported that *TOMM40* pseudogene transcripts are expressed across different tissue types in both cancer and non-cancer cells [69]. Pseudogene-derived transcripts compete for gene regulatory and post-transcriptional regulatory elements [69]. Therefore, polymorphisms that contribute to differences between *TOMM40* and its pseudogene may result in changes in enhancer activities and promoter interactions of the gene networks.

## Limitations

Our literature review attempted to include research topics associated with *TOMM40*, healthy aging, and longevity by searching the key words across all fields; but not solely on article titles and abstracts. We used broad terminologies such as “Aging” and “Longevity” in hoping to encompass most existing studies associated with aging-related phenotypes and human longevity. However, such broad terms may result in failure to capture research studies that used very specific keywords that are related to mental or physical disabilities related to the elderly. Approximately two-third of the studies identified in the systematic review investigated into very specific *TOMM40*, or identify SNP of interest using commercially available SNP arrays. This may contribute to an overemphasis of specific *TOMM40* SNP. Additionally, because the remaining one-third of the studies were GWAS studies, many did not propose clear biological mechanisms that could explain how the SNPs could be tied to longevity or healthy aging.

It has caught our attention that most longevity studies simply compared the chronological ages between participants of different genetic haplotypes, or simply set the cutoff point (i.e., 90 years old) for longevity. Current studies investigating the association between *TOMM40* and longevity have not accounted for healthy aging (i.e., changes in mental and/or physical capabilities due to aging progress). Although people from a specific *TOMM40* genetic subgroup may have a longer lifespan, it is not known whether these people also have a longer HALE (i.e., having lesser or delayed onset of disabilities), or they simply have a longer life expectancy. We recommend that future studies should also take years of a healthy life into account to determine if the study participants truly lived a long and healthy life.

## Conclusions

Additional studies are recommended to ascertain the relationship of *TOMM40* genetic variations with other chronic conditions, number of comorbidities, and frailty. Although our review did not consider Alzheimer’s dementia or other diseases, it is possible that the same variations have effects on both disease and healthy aging. Considering disease states could elucidate not just the underlying mechanisms behind longevity, but also provide insights on increasing healthy life expectancy.

The five candidate SNPs discussed in this paper can become potential candidates to be examined together for future studies on aging-associated comorbidities. Aside from the commonly investigated SNPs rs10524523 and rs2075650, there are currently no reports on how the other three candidates (rs71352238, rs157582, and rs8106922) can influence *TOMM40* RNA or protein level. Furthermore, researchers have yet to find the biological implication of rs8106922 and have not postulated any specific mechanisms that can tie to increased longevity. Thus, functional studies of the 5 intronic SNPs (rs10524523, rs2075650, rs71352238, rs157582, and rs8106922) as well as gene sequencing of *TOMM40* are warranted.

Additional work is required to ascertain how these SNP candidates can affect inter-gene and intra-gene regulations, RNA expression, and protein expression. Future research direction should also focus on examining the intronic SNPs and their associations with alternative splicing of truncated TOM40 proteins. These investigations can identify the potential biological significance of the polymorphisms and protein variances, and help devise potential therapies that can improve the health of individuals who did not inherit longevity alleles to become more resilient against age-related disabilities. These researches will improve the overall quality of life, and decrease the gap between healthy life expectancy and life expectancy.

## Data Availability

All data generated or analyzed during this study are included in this published article.

## Abbreviations

AD: Alzheimer’s Disease
BMI: Body Mass Index
HALE: Healthy Life Expectancy
mtDNA: Mitochondrial DNA
SNPs: Single Nucleotide Polymorphisms
TOM: Translocase of Mitochondrial Outer Membrane
VRF: Vascular Risk Factor
